# Effects of finger acupressure combined with lower limb rehabilitation training machine on stroke recovery

**DOI:** 10.3389/fneur.2025.1609815

**Published:** 2025-08-22

**Authors:** Xiaoxue Liu, Feng Zhang, Yanhong Li, Jieqiong Zhao, Yatao Du, Qian Zhang, Weifang Li

**Affiliations:** ^1^Rehabilitation Department of Hebei Province Traditional Chinese Medicine Hospital, Shijiazhuang, China; ^2^Rehabilitation Department of the Third Hospital of Hebei Medical University, Shijiazhuang, China; ^3^Rehabilitation Department of Hebei University of Chinese Medicine Fourth Affiliated Hospital, Shijiazhuang, China; ^4^Rehabilitation Department of Medicine, Xingtai People’s Hospital, Xingtai, China

**Keywords:** finger acupressure, lower limb rehabilitation training machine, stroke, motor function, balance ability

## Abstract

**Background:**

Stroke is a common acute cerebrovascular disease, and rehabilitation therapy plays a crucial role in the recovery of stroke patients.

**Methods:**

In this retrospective study, we first enrolled 80 stroke patients. These participants were then randomly divided into two groups: the treatment group underwent finger acupressure combined with lower limb rehabilitation training machine, and the control group received basic rehabilitation therapy. Baseline differences between the two groups were analyzed, as well as changes in motor function (FMA, sFMA, MAS, 10-Meter Walk Test, 6-Minute Walk Test), balance ability (Tinetti Test, FRT, MRT, TUGT), neural repair markers (NSE, NFL, BDNF), blood and gas exchange parameters (SpO₂, RDW, PLT), and immune and inflammatory responses (CRP, IL-6, IL-10) before and after rehabilitation. A multivariate logistic regression analysis was conducted to evaluate the impact of rehabilitation methods, stroke-related factors, and their interactions on motor function and balance recovery. Additionally, long-term quality of life and one-year recurrence rates were compared between the treatment and control groups.

**Results:**

After treatment, compared with the control group, the treatment group showed significant improvements in motor function, balance ability, nerve repair, blood and gas exchange, immune and inflammatory response indicators. In addition to treatment methods, stroke type, stroke location, NIHSS score, and mRS score also significantly affect the recovery of motor function and balance ability. The treatment group has a better therapeutic effect in patients with lower baseline NIHSS scores, mRS scores, and ischemic stroke. The combination of finger acupressure and lower limb rehabilitation training machines can significantly improve the long-term quality of life of patients and reduce the one-year stroke recurrence rate.

**Conclusion:**

Finger acupressure combined with the lower limb rehabilitation training machine enhances motor function and balance recovery in stroke patients by promoting neural repair, improving immune function, and reducing inflammatory responses. This therapeutic approach is particularly effective in patients with lower baseline NIHSS and mRS scores and those with ischemic stroke.

## 1 Introduction

“Cerebral stroke” also known as “stroke” or “cerebrovascular accident (CVA),” is an acute cerebrovascular disease (1). It is a group of diseases caused by sudden rupture of cerebral blood vessels or blockage of blood vessels, leading to impaired blood flow to the brain and resulting in brain tissue damage (2). Stroke is characterized by high incidence, high mortality, and high disability rates. It includes ischemic and hemorrhagic strokes, with ischemic stroke being more common, accounting for 60–70% of all strokes (3, 4). Internal carotid artery and vertebral artery occlusion and stenosis can lead to ischemic stroke, mostly occurring in individuals over 40 years old, with a higher incidence in males than females (5, 6). Severe cases may result in death. Hemorrhagic stroke has a higher mortality rate (7). Studies indicate that stroke has become the leading cause of death in China and the primary cause of disability among Chinese adults (8).

Patients with stroke experience significant declines in motor function and balance ability, which severely impact their quality of life and independence (9–11). Rehabilitation therapy plays a crucial role in the recovery process of stroke patients. Traditional stroke rehabilitation primarily includes physical therapy, occupational therapy, and speech therapy (12). With the development of rehabilitation medicine, finger acupressure and lower limb rehabilitation training machine have emerged as new rehabilitation methods and have been increasingly applied in clinical practice. Lower limb rehabilitation training machine, also known as exoskeletal lower limb walking rehabilitation robots, are primarily used for rehabilitation training in patients with walking impairments due to brain, spinal cord, muscle, or skeletal diseases (13). Combining this with traditional Chinese medicine finger acupressure can promote neural pathway reconstruction in stroke patients, effectively enhancing their walking ability and ultimately aiding in the recovery of motor function, enabling patients to return to their families and society as soon as possible.

Existing studies have shown that both acupressure and robot-assisted upper and lower limb rehabilitation contribute to the motor recovery of stroke patients finger acupressure (14–16), but few studies have combined the two methods. This study aims to analyze the effects of finger acupressure combined with a lower limb rehabilitation training machine on the recovery of motor function and balance ability in stroke patients. The findings will provide an effective rehabilitation treatment plan for improving stroke patients’ walking function. The impact of the interaction between rehabilitation therapy methods and stroke-related factors on the recovery of stroke patients was also evaluated, which was not covered in previous studies. This study offers objective evidence for rehabilitation physicians in understanding stroke walking function recovery and prognosis. It will provide objective indicators for integrating traditional Chinese medicine treatment with modern medical equipment.

## 2 Materials and methods

### 2.1 Patient selection

This retrospective study included stroke patients who were hospitalized in our hospital from September 2022 to June 2023. Patients were diagnosed with stroke based on medical history, imaging examinations, and clinical manifestations, meeting the relevant clinical diagnostic criteria outlined in the “2022 Stroke Diagnosis and Treatment Guidelines.” The inclusion criteria are: meeting the diagnostic criteria for stroke mentioned above; Patients who require rehabilitation treatment and are willing to receive finger acupressure combined with the lower limb rehabilitation training machine. The exclusion criteria are: cases that have been treated with other treatments since the onset of the disease and those who are currently participating in other clinical trials; Having serious primary heart, nervous system, blood or other serious diseases that affect their survival; Interrupt the participant on their own; Those who have not been treated according to regulations, are unable to determine the efficacy, or have incomplete data that affects the assessment of efficacy; Patients with lower limb fractures or cognitive impairments who are unable to cooperate. Computer-generated random numbers were used to randomly select 40 patients from each group, with a total of 80 patients, to ensure that each group’s sample was representative and to reduce bias.

### 2.2 Treatment methods

The control group received basic rehabilitation treatment, including physical therapy, occupational therapy, and speech therapy. Physical therapy included exercise therapy, electrotherapy, and thermal/cold therapy. Occupational therapy included functional training, cognitive training, and psychological training. Treatment was conducted 3–5 times per week, for 30–45 min per session, over 8 weeks.

The treatment group received finger acupressure combined with lower limb rehabilitation training machine therapy in addition to basic rehabilitation treatment. Finger acupressure was applied to the following acupressure: Zusanli (ST36), Xuehai (SP10), Sanyinjiao (SP6), Yanglingquan (GB34), Feishu (BL13), Pishu (BL20), Shenshu (BL23), Fenglong (ST40), and Taichong (LR3). Each session lasted 20 min, conducted continuously for 5 days per cycle. The lower limb rehabilitation training machine was used 2–3 times per day, for 20–30 min per session, 5 days per cycle. The treatment lasted for eight cycles, with each cycle lasting 7 days.

### 2.3 Data collection

Collect baseline information of patients, including their age, gender BMI, Hypertension, diabetes, heart disease, stroke type (ischemic stroke or hemorrhagic stroke), stroke site (left hemisphere, right hemisphere or brain stem), whether to receive thrombolysis treatment, mechanical thrombectomy or surgical treatment, mRS score (mild: 0–2, moderate: 3, severe: 4–5), NIHSS score (mild: 1–4, moderate: 5–15, severe: 16–20, very severe: 21–24). And before and after rehabilitation treatment, FMA (Fugl Meyer Assessment), upper limb FMA (FMA-UE), lower limb FMA (FMA-LE), MAS (Modified Ashworth Scale), simplified FMA (sFMA), mRS (Modified Rankin Scale), Tinetti balance and gait assessment (Tinetti Test), modified Romberg test (MRT, assessing moderate and severe instability), Functional Reach Test (FRT), TUGT (Timed Up and Go Test), 10 meter walk test (10MWT), and 6-min walk test (6MWT). NSE (Neuron-Specific Enolase), NFL (Neurofilament light), BDNF (Brain-Derived Neurotrophic Factor), SpO₂ (Peripheral Capillary Oxygen Saturation), RDW (Red Cell Distribution Width), PLT (Platelet Count), CRP (C-Reactive Protein), IL-10 (Interleukin-10), and IL-6 (Interleukin-6). Collect the occurrence of adverse events during treatment. At the completion of treatment, 1 month after treatment, and 3 months after treatment, quality of life scores (Stroke Specific Quality of Life, SS-QoL) were collected, as well as information on stroke recurrence within 1 year.

After treatment, if the FMA score increases by ≥ 10 points compared to baseline, the sFMA score increases by ≥ 5 points, muscle tone decreases by at least 1 level, walking speed increases by ≥ 0.16 m/s, walking distance increases by ≥ 50 meters, and four or more indicators meet the above criteria, it can be judged as improvement in motor function. Before and after treatment, the Tinetti Test score increased by ≥ 5 points, the FRT extension distance increased by ≥ 5 cm, the MRT standing stability score increased by ≥ 1 level, and the TUGT completion time was shortened by ≥ 3 s. If three or more indicators meet the above criteria, it can be defined as an improvement in balance ability.

### 2.4 Data analysis

All analyses were performed using R 4.4.0 software. Continuous data were expressed as median (minimum-maximum) and were analyzed using independent sample t-tests or Mann–Whitney U tests. Categorical data were presented as frequencies (percentages) and were analyzed using chi-square tests or Fisher’s exact tests. Before conducting parametric or non-parametric tests, normality tests and homogeneity of variance tests should be performed first. If both normal distribution and homogeneity of variance are met, parametric tests should be used; otherwise, non-parametric tests should be employed. Multivariate logistic regression analysis was used to assess the effects of rehabilitation treatment methods, stroke-related factors, and their interaction on the recovery of motor function and balance ability. All statistical tests were two-tailed, with *p* < 0.05 considered statistically significant.

## 3 Results

### 3.1 Baseline information of stroke patients

The cohort comprised 80 stroke patients (55% male) with median age 55 years (range 32–77) and BMI 28.4 (23.7–32.7). Baseline characteristics including comorbidities (hypertension 11.3%, diabetes 8.8%, heart disease 16.3%), stroke types (ischemic 72.5%), lesion locations, and treatment modalities were well-balanced between groups (all *p* > 0.05, Table 1). Functional status at enrollment showed 60% of patients had an mRS score of 0–2, while NIHSS scores indicated 42.5% with mild stroke severity (Table 1).

**Table 1:** Demographic and clinical information of stroke patients.

Variables	All patients (*n* = 80)	Control group (*n* = 40)	Treatment group (*n* = 40)	*p*-value
Age	55 (32–77)	54 (33–74)	56 (32–77)	0.977
Gender				0.26115
Male	44 (55%)	25 (62.5%)	19 (47.5%)	
Female	36 (45%)	15 (37.5%)	21 (52.5%)	
BMI	28.4 (23.7–32.7)	28.3 (23.7–32.7)	28.4 (24.0–32.7)	0.855
Hypertension				0.15697
Yes	9 (11.25%)	2 (5%)	7 (17.5%)	
No	71 (88.75%)	38 (95%)	33 (82.5%)	
Diabetes mellitus				0.11349
Yes	7 (8.75%)	6 (15%)	1 (2.5%)	
No	73 (91.25%)	34 (85%)	39 (97.5%)	
Heart disease				0.22541
Yes	13 (16.25%)	9 (22.5%)	4 (10%)	
No	67 (83.75%)	31 (77.5%)	36 (90%)	
Type of stroke				0.21059
Ischemic stroke	58 (72.5%)	32 (80%)	26 (65%)	
Hemorrhagic stroke	22 (27.5%)	8 (20%)	14 (35%)	
Stroke location				0.63674
Left hemisphere	36 (45%)	16 (40%)	20 (50%)	
Right hemisphere	32 (40%)	17 (42.5%)	15 (37.5%)	
Brainstem	12 (15%)	7 (17.5%)	5 (12.5%)	
Thrombolysis				0.31049
Yes	10 (12.5%)	7 (17.5%)	3 (7.5%)	
No	70 (87.5%)	33 (82.5%)	37 (92.5%)	
Mechanical thrombectomy				0.08567
Yes	15 (18.75%)	11 (27.5%)	4 (10%)	
No	65 (81.25%)	29 (72.5%)	36 (90%)	
Surgery				0.47392
Yes	2 (2.5%)	0 (0%)	2 (5%)	
No	78 (97.5%)	40 (100%)	38 (95%)	
mRS, Modified Rankin Scale				0.05167
Mild (0–2)	48 (60%)	24 (60%)	24 (60%)	
Moderate (3)	27 (33.75%)	11 (27.5%)	16 (40%)	
Severe (4–5)	5 (6.25%)	5 (12.5%)	0 (0%)	
NIHSS, National Institutes of Health Stroke Scale				0.4045
Mild (1–4)	34 (42.5%)	18 (45%)	16 (40%)	
Moderate (5–15)	30 (37.5%)	16 (40%)	14 (35%)	
Severe (16–20)	10 (12.5%)	5 (12.5%)	5 (12.5%)	
Very Severe (21–24)	6 (7.5%)	1 (2.5%)	5 (12.5%)	

### 3.2 Differences in motor function and balance ability indicators between the treatment and control groups before and after rehabilitation

Before rehabilitation treatment, there were no significant differences in motor function and balance ability indicators (such as FMA, MAS, TUGT, etc.) between the treatment and control groups. After treatment, the treatment group showed significantly higher FMA-UE, FMA-LE, sFMA, Tinetti Test, FRT, and 6MWT scores than the control group, while MAS, mRS, MRT, TUGT, and 10MWT scores were significantly lower in the treatment group compared to the control group. This indicates that the treatment group had significantly better recovery in terms of motor function, balance ability, and gait assessment (Table 2).

**Table 2:** Differences in motor function and balance ability indicators between the control group and treatment group before and after rehabilitation training.

Variables	All patients (*n* = 80)	Control group (*n* = 40)	Treatment group (*n* = 40)	*p*-value
FMA, Fugl-Meyer Assessment				
FMA-UE				
Before rehabilitation treatment	29 (15–41)	28 (16–41)	30 (15–41)	0.744
After rehabilitation treatment	44 (27–61)	42 (27–59)	47 (27–61)	0.0311
FMA-LE				
Before rehabilitation treatment	16 (8–23)	16 (8–22)	16 (8–23)	0.765
After rehabilitation treatment	21 (12–30)	20 (12–28)	23 (12–30)	0.0319
MAS, Modified Ashworth Scale				
Before rehabilitation treatment	2 (1–4)	3 (1–4)	2 (1–4)	0.181
After rehabilitation treatment	2 (0–3)	2 (0–3)	1 (0–3)	0.0237
sFMA, Simplified Fugl-Meyer Assessment				
Before rehabilitation treatment	19 (12–25)	19 (12–25)	19 (12–24)	0.63
After rehabilitation treatment	29 (19–39)	27 (19–39)	30 (20–39)	0.0276
mRS, Modified Rankin Scale				
Before rehabilitation treatment	2 (0–5)	2 (0–5)	2 (0–5)	0.465
After rehabilitation treatment	2 (0–4)	2 (0–4)	1 (0–4)	0.0243
Tinetti Balance and Gait Assessment, Tinetti Test				
Before rehabilitation treatment	15 (8–21)	15 (8–21)	15 (8–21)	0.802
After rehabilitation treatment	22 (15–27)	20 (15–27)	23 (15–27)	0.0157
Modified Romberg Test, MRT (proportion of moderate and severe instability)				
Before rehabilitation treatment	52%	51%	53%	0.303
After rehabilitation treatment	12%	14%	10%	0.0289
Functional Reach Test, FRT (cm)				
Before rehabilitation treatment	24 (21–27)	24 (21–27)	23 (21–27)	0.392
After rehabilitation treatment	33 (28–40)	32 (28–39)	35 (29–40)	0.00111
TUGT, Timed Up and Go Test (s)				
Before rehabilitation treatment	24 (16–33)	23 (16–33)	25 (16–33)	0.758
After rehabilitation treatment	11 (4–17)	14 (5–17)	9 (4–16)	0.000105
10-Meter Walk Test, 10MWT (s)				
Before rehabilitation treatment	19 (12–26)	20 (12–26)	19 (13–26)	0.538
After rehabilitation treatment	10 (5–16)	12 (5–16)	9 (5–16)	0.0127
6-Minute Walk Test, 6MWT				
Before rehabilitation treatment	174 (119–230)	175 (121–230)	169 (119–230)	0.693
After rehabilitation treatment	401 (256–550)	352 (259–537)	434 (256–550)	0.00481

### 3.3 Differences in physiological and biochemical indicators between the treatment and control groups before and after rehabilitation

Before rehabilitation treatment, there were no significant differences in various neurological injury and repair-related indicators, inflammation-related indicators, and blood-related indicators between the treatment and control groups. After treatment, the treatment group showed significantly higher levels of BDNF (Brain Derived Neurotrophic Factor), SpO2 (Blood Oxygen Saturation), RDW (Red Blood Cell Distribution Width), and IL-10 (Interleukin-10) compared to the control group. In contrast, the treatment group showed significantly lower levels of PLT (Platelet Count), CRP (C-reactive Protein), and IL-6 (Interleukin-6) compared to the control group (Table 3). This suggests that the combination of finger acupressure and lower limb rehabilitation training machine had a significant positive impact on patients’ neurorepair, immune function, and inflammatory responses.

**Table 3:** Differences in physiological and biochemical indicators between the control group and treatment group before and after rehabilitation treatment.

Variables	All patients (*n* = 80)	Control group (*n* = 40)	Treatment group (*n* = 40)	*p*-value
NSE, Neuron-Specific Enolase (ng/mL)				
Before rehabilitation treatment	20.38 (13.51–26.76)	20.37 (13.79–26.13)	20.46 (13.51–26.76)	0.43
After rehabilitation treatment	12.70 (8.37–17.26)	13.93 (10.35–17.26)	11.49 (8.37–16.63)	0.000757
NFL, Neurofilament light (pg/mL)				
Before rehabilitation treatment	150.00 (110.31–195.19)	155.91 (110.31–194.49)	145.51 (120.36–195.19)	0.637
After rehabilitation treatment	66.86 (52.99–81.45)	71.83 (57.66–81.45)	61.95 (52.99–78.91)	9.73E-05
BDNF, Brain-Derived Neurotrophic Factor				
Before rehabilitation treatment	7.49 (4.51–10.79)	7.49 (4.55–10.74)	7.39 (4.51–10.79)	0.977
After rehabilitation treatment	18.95 (9.49–31.66)	14.69 (9.49–29.42)	24.57 (10.09–31.66)	0.00378
SpO2, Oxygen Saturation (%)				
Before rehabilitation treatment	93.05 (91.12–95.35)	92.80 (91.23–95.34)	93.20 (91.12–95.35)	0.686
After rehabilitation treatment	97.10 (95.24–98.79)	96.81 (95.24–98.79)	97.64 (95.28–98.75)	0.0304
Red Cell Distribution Width, RDW (%)				
Before rehabilitation treatment	18.23 (15.74–20.00)	18.04 (16.04–19.92)	18.46 (15.74–20.00)	0.57
After rehabilitation treatment	12.96 (11.28–15.25)	13.70 (11.29–15.23)	12.66 (11.28–15.25)	0.0166
Platelet Count, PLT (× 10^9^/L)				
Before rehabilitation treatment	441.49 (353.29–513.52)	446.26 (353.29–513.52)	434.27 (353.76–512.95)	0.744
After rehabilitation treatment	280.19 (181.43–409.24)	312.49 (208.65–409.24)	253.44 (181.43–404.41)	0.00244
CRP, C-Reactive Protein (mg/L)				
Before rehabilitation treatment	26.01 (18.55–35.96)	26.86 (19.19–35.54)	25.16 (18.55–35.96)	0.465
After rehabilitation treatment	7.82 (4.33–12.31)	9.45 (4.33–12.31)	7.12 (4.44–11.37)	0.000286
IL-10, Interleukin-10 (pg/mL)				
Before rehabilitation treatment	23.65 (13.52–31.90)	22.70 (14.06–31.90)	25.29 (13.52–31.90)	0.482
After rehabilitation treatment	7.03 (3.62–9.58)	6.54 (3.62–9.53)	7.94 (4.12–9.58)	0.00453
IL-6, Interleukin-6 (pg/mL)				
Before rehabilitation treatment	20.29 (13.21–28.60)	20.50 (13.21–28.60)	20.23 (13.31–28.29)	0.817
After rehabilitation treatment	5.63 (3.20–8.58)	6.63 (3.20–8.58)	4.91 (3.21–8.19)	0.0026

### 3.4 Differences in adverse event rates between the treatment and control groups during treatment

In the treatment group, 22.5% of patients reported muscle or joint discomfort, while in the control group, 7.5% of patients reported the same. There was no significant difference between the two groups (*p* = 0.117). In the treatment group, 22.5% of patients reported fatigue, while 12.5% in the control group reported fatigue. There was no significant difference between the two groups (*p* = 0.377). In the treatment group, 10% of patients reported skin damage, while 5% in the control group reported the same. There was no significant difference between the two groups (*p* = 0.671). In the control group, 12.5% of patients reported blood pressure fluctuations, while no such issues were reported in the treatment group. This was close to statistical significance (*p* = 0.065). In the treatment group, 15% of patients reported mood swings or anxiety, while 7.5% in the control group reported the same. There was no significant difference between the two groups (*p* = 0.479). Overall, there was no significant difference in the adverse event rates between the treatment and control groups (Table 4).

**Table 4:** The incidence of adverse events during treatment in the control group and treatment group.

Adverse events	All patients (*n* = 80)	Control group (*n* = 40)	Treatment group (*n* = 40)	*p*-value
Muscle or joint discomfort				0.1174515
Yes	12 (15%)	3 (7.5%)	9 (22.5%)	
No	68 (85%)	37 (92.5%)	31 (77.5%)	
Fatigue				0.3773796
Yes	14 (17.5%)	5 (12.5%)	9 (22.5%)	
No	66 (82.5%)	35 (87.5%)	31 (77.5%)	
Skin damage				0.6712184
Yes	6 (7.5%)	2 (5%)	4 (10%)	
No	74 (92.5%)	38 (95%)	36 (90%)	
Blood pressure fluctuations				0.0646717
Yes	5 (6.25%)	5 (12.5%)	0 (0%)	
No	75 (93.75%)	35 (87.5%)	40 (100%)	
Emotional fluctuations or anxiety				0.4791565
Yes	9 (11.25%)	3 (7.5%)	6 (15%)	
No	71 (88.75%)	37 (92.5%)	34 (85%)	

### 3.5 Multivariate logistic regression analysis of the effects of stroke-related factors and rehabilitation treatment methods on motor function recovery in stroke patients

The results showed that the estimated value of the treatment method was 0.340, with a *p* value less than 0.001, and an OR (Odds Ratio) of 1.405 (95% CI: 1.190–1.659), indicating that the combination of finger acupressure and lower limb rehabilitation training machine significantly improved patients’ motor function. Besides rehabilitation methods, stroke-related factors also significantly influenced motor function recovery in stroke patients. The estimated value for left hemisphere stroke was 0.295, with a *p* value of 0.019, and an OR of 1.343 (95% CI: 1.056–1.709), indicating that patients with left hemisphere stroke had better motor function improvement compared to those with brainstem stroke. The estimated value for right hemisphere stroke was 0.455, with a *p* value less than 0.001, and an OR of 1.576 (95% CI: 1.234–2.012), indicating that patients with right hemisphere stroke showed more significant improvement in motor function compared to those with brainstem stroke. The estimated value for NIHSS score was −0.022, with a *p* value of 0.003, and an OR of 0.978 (95% CI: 0.964–0.992), suggesting that the higher the baseline NIHSS score, the lower the possibility of motor function improvement. The estimated value for mRS score was −0.160, with a *p* value of 0.001, and an OR of 0.852 (95% CI: 0.778–0.933), indicating that the higher the baseline mRS score, the lower the possibility of motor function improvement. We also conducted an interaction analysis to determine which patients benefited most from the rehabilitation treatment method. The results showed significant interactions between rehabilitation treatment methods and both NIHSS and mRS scores, with negative B values (−0.431 and −0.651, respectively), indicating that the combination of finger acupressure and lower limb rehabilitation training machine was less effective in patients with higher baseline NIHSS and mRS scores. However, the location of stroke had no significant effect on the intervention’s efficacy (Table 5).

**Table 5:** Multivariate logistic regression analysis of stroke-related factors and rehabilitation methods on motor function in stroke patients.

Term	Estimate	std.error	Statistic	*p* value	OR	CI-lower	CI-upper
Treatment method	0.340	0.085	4.007	0.000	1.405	1.190	1.659
Type of stroke (ischemic stroke)	−0.066	0.092	−0.711	0.479	0.936	0.781	1.122
Stroke location (left hemisphere)	0.295	0.123	2.400	0.019	1.343	1.056	1.709
Stroke location (right hemisphere)	0.455	0.125	3.645	0.000	1.576	1.234	2.012
NIHSS	−0.022	0.007	−3.084	0.003	0.978	0.964	0.992
mRS	−0.160	0.046	−3.467	0.001	0.852	0.778	0.933
Type of stroke (ischemic stroke)	−0.078	0.093	−0.841	0.403	0.925	0.771	1.109
NIHSS	−0.022	0.007	−3.022	0.003	0.978	0.965	0.992
mRS	−0.167	0.046	−3.602	0.001	0.846	0.772	0.926
Treatment method	0.593	0.212	2.798	0.007	1.810	1.194	2.743
Stroke location (left hemisphere)	0.395	0.160	2.473	0.016	1.485	1.085	2.031
Stroke location (right hemisphere)	0.622	0.171	3.645	0.001	1.863	1.333	2.603
Treatment method*stroke location (left hemisphere)	−0.248	0.245	−1.010	0.316	0.781	0.483	1.262
Treatment method*stroke location (right hemisphere)	−0.360	0.251	−1.438	0.155	0.697	0.427	1.140
Type of stroke (ischemic stroke)	−0.041	0.098	−0.420	0.675	0.960	0.793	1.162
Stroke location (left hemisphere)	0.307	0.124	2.471	0.016	1.359	1.065	1.733
Stroke location (right hemisphere)	0.456	0.125	3.643	0.001	1.577	1.234	2.015
mRS	−0.154	0.047	−3.289	0.002	0.857	0.782	0.940
Treatment method	0.201	0.196	1.026	0.308	1.223	0.833	1.796
NIHSS	−0.027	0.009	−2.848	0.006	0.973	0.955	0.992
Treatment method*NIHSS	−0.431	0.211	−2.046	0.041	0.650	0.430	0.982
Type of stroke (ischemic stroke)	−0.060	0.093	−0.652	0.517	0.941	0.785	1.129
Stroke location (left hemisphere)	0.306	0.124	2.472	0.016	1.358	1.065	1.731
Stroke location (right hemisphere)	0.469	0.126	3.721	0.000	1.598	1.248	2.046
NIHSS	−0.022	0.007	−3.013	0.004	0.978	0.965	0.992
Treatment method	0.135	0.250	0.539	0.592	1.144	0.701	1.869
mRS	−0.207	0.071	−2.924	0.005	0.813	0.708	0.934
Treatment method*mRS	−0.651	0.281	−2.320	0.020	0.522	0.301	0.904

### 3.6 Multivariate logistic regression analysis of the effects of stroke-related factors and rehabilitation treatment methods on balance recovery in stroke patients

The results indicated that the estimated value for rehabilitation treatment was 0.387 (*p* < 0.001), showing that patients receiving the combination of finger acupressure and lower limb rehabilitation training machine had significantly better balance recovery than those receiving basic treatment, with an OR of 1.473 (95% CI: 1.223–1.773). The estimated value for stroke type was 0.307 (*p* = 0.003), suggesting that patients with ischemic stroke had better balance recovery, with an OR of 1.359 (95% CI: 1.120–1.648). NIHSS and mRS scores were significantly negatively correlated with balance recovery, with estimated values of −0.018 (*p* = 0.023) and −0.147 (*p* = 0.008), respectively. Interaction analysis revealed a significant positive correlation between rehabilitation treatment methods and stroke type for balance recovery. That is, patients with ischemic stroke who received the combination of finger acupressure and lower limb rehabilitation training machine had better balance recovery (Table 6).

**Table 6:** Multivariate logistic regression analysis of stroke-related factors and rehabilitation methods on balance recovery in stroke patients.

Term	Estimate	std.error	Statistic	*p* value	OR	CI-lower	CI-upper
Treatment method	0.387	0.095	4.088	0.000	1.473	1.223	1.773
Type of stroke (ischemic stroke)	0.307	0.099	3.112	0.003	1.359	1.120	1.648
Stroke location (left hemisphere)	0.085	0.134	0.637	0.526	1.089	0.837	1.417
Stroke location (right hemisphere)	0.234	0.131	1.777	0.080	1.263	0.976	1.634
NIHSS	−0.018	0.008	−2.326	0.023	0.982	0.967	0.997
mRS	−0.147	0.053	−2.749	0.008	0.863	0.777	0.959
Stroke location (left hemisphere)	0.080	0.134	0.601	0.550	1.084	0.834	1.408
Stroke location (right hemisphere)	0.250	0.131	1.906	0.061	1.284	0.993	1.661
mRS	−0.158	0.054	−2.938	0.004	0.854	0.768	0.949
NIHSS	−0.018	0.008	−2.311	0.024	0.982	0.967	0.997
Treatment method	0.188	0.175	1.077	0.285	1.207	0.857	1.701
Type of stroke (ischemic stroke)	0.162	0.145	1.114	0.269	1.176	0.884	1.563
Treatment method*type of stroke (ischemic stroke)	0.488	0.082	5.955	0.000	1.628	1.387	1.912
Stroke location (left hemisphere)	0.085	0.137	0.624	0.535	1.089	0.833	1.424
Stroke location (right hemisphere)	0.233	0.135	1.730	0.088	1.263	0.969	1.645
Type of stroke (ischemic stroke)	0.306	0.099	3.084	0.003	1.359	1.118	1.651
mRS	−0.147	0.054	−2.726	0.008	0.863	0.777	0.960
Treatment method	0.388	0.209	1.860	0.067	1.475	0.979	2.221
NIHSS	−0.018	0.011	−1.713	0.091	0.982	0.962	1.003
Treatment method*NIHSS	0.000	0.016	−0.008	0.994	1.000	0.970	1.031
Stroke location (left hemisphere)	0.072	0.134	0.536	0.593	1.075	0.826	1.398
Stroke location (right hemisphere)	0.204	0.133	1.529	0.131	1.226	0.944	1.591
Type of stroke (ischemic stroke)	0.294	0.099	2.984	0.004	1.342	1.106	1.629
NIHSS	−0.018	0.008	−2.304	0.024	0.982	0.967	0.997
Treatment method	0.088	0.260	0.337	0.737	1.092	0.656	1.817
mRS	−0.207	0.072	−2.869	0.005	0.813	0.705	0.936
Treatment method*mRS	0.121	0.098	1.236	0.220	1.129	0.932	1.368

### 3.7 Differences in Long-term quality of life and stroke recurrence rates within 1 year between the treatment and control groups after rehabilitation treatment

**Figure 1:**
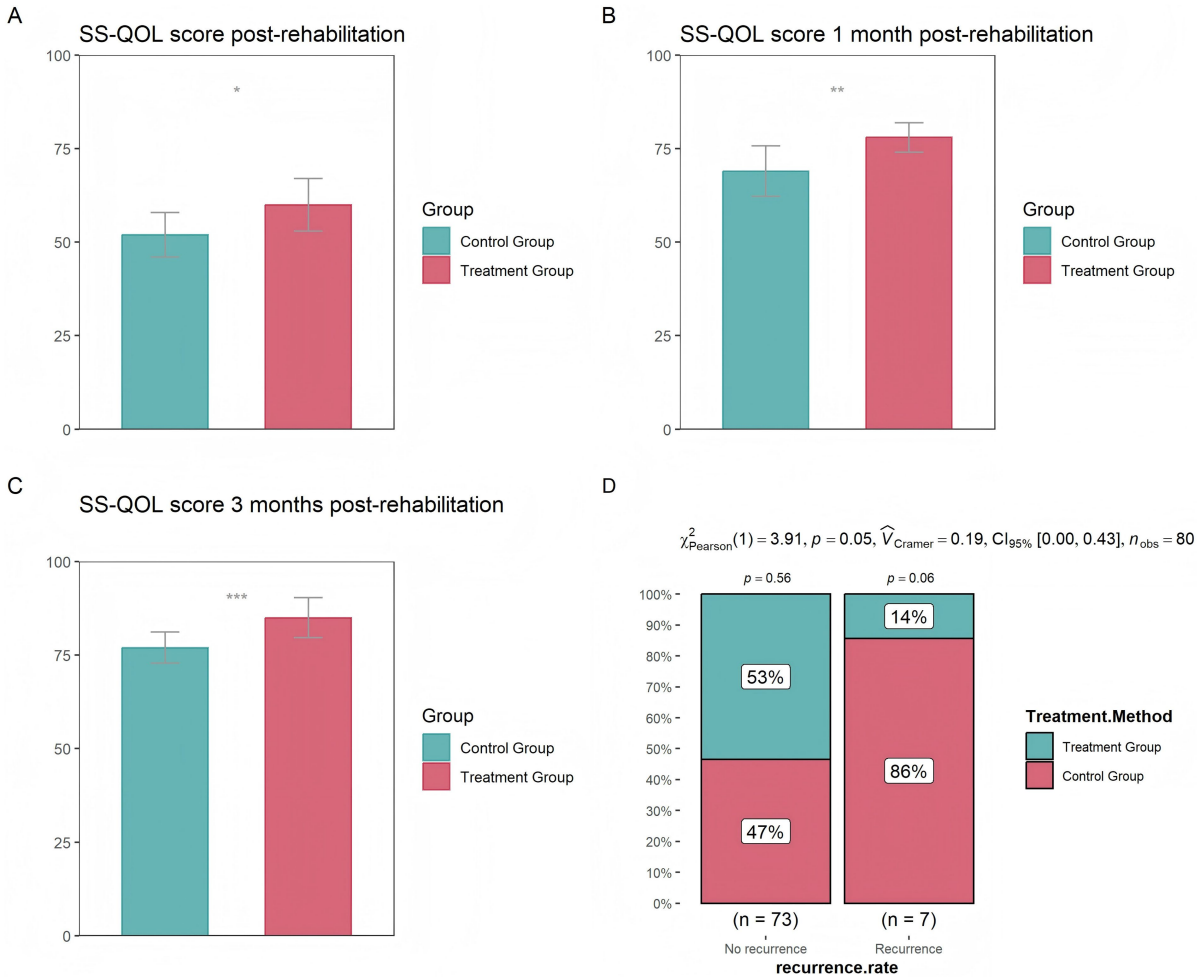
Differences in quality of life between the control and treatment groups **(A)** at the completion of treatment **(B)** 1 month after treatment **(C)** 3 months after treatment **(D)** Differences in stroke recurrence rates between the control and treatment groups over the course of 1 year (* *p* < 0.05, ** *p* < 0.01, *** *p* < 0.001).

The results indicated that, immediately after rehabilitation treatment, and at 1 month and 3 months after rehabilitation treatment, the treatment group had significantly higher quality of life scores than the control group, with the most significant difference observed at 3 months (Figures 1A–C). In terms of recurrence rates within 1 year, the proportion of patients with stroke recurrence in the treatment group was significantly lower than that of patients without recurrence (14% vs. 53%) (Figure 1D), further demonstrating that the treatment group had a lower stroke recurrence rate.

## 4 Discussion

This study indicates that finger acupressure combined with lower limb rehabilitation training machine is closely related to the improvement of motor function and balance ability in stroke patients. Further physiological and biochemical indicators revealed that after treatment, the treatment group showed significantly higher levels of BDNF, SpO2, RDW, and IL-10 compared to the control group. However, in terms of PLT, CRP, IL-6 and other indicators, the treatment group was significantly lower than the control group. BDNF is an important neurotrophic factor that primarily functions to promote neuronal survival, enhance synaptic plasticity, and thus facilitate nerve injury recovery (17, 18). The finger acupressure combined with the lower limb rehabilitation machine provides specific stimuli to body parts, activating reflex zones and specific pathways such as the PI3K/Akt and MAPK/ERK pathways, thus increasing the transcription and secretion of BDNF. Neurofilament light (NFL) is an important component of the neuronal cytoskeleton and belongs to the intermediate filaments family (19). After stroke, ischemia or hemorrhage leads to neuronal death and axonal breakage, and neurofilament proteins are released from damaged neurons into cerebrospinal fluid and blood, thus directly reflecting the extent of neuronal damage (20). Neuron-specific enolase (NSE) is a glycolytic enzyme mainly present in neurons and neuroendocrine cells. It is one of the isoenzymes of enolase and is also regarded as a biomarker of neural injury (21, 22). Finger acupressure activates the somatosensory-motor cortex neural projection pathway by stimulating specific acupoints, promoting the release of neurotrophic substances such as brain-derived neurotrophic factor (BDNF), enhancing synaptic plasticity, and accelerating axonal regeneration, thereby repairing damaged neural connections. Meanwhile, lower limb rehabilitation training machines strengthen the “motor-sensory feedback loop” through repetitive motor training, promoting cortical spinal cord functional reorganization and neural pathway remodeling. This dual intervention not only significantly enhances the plasticity of the nervous system but also, more importantly, effectively reduces the extent of neuronal damage by reducing neuronal apoptosis caused by ischemia and hypoxia, thereby decreasing the levels of NFL and NSE.

We also found that the combination of finger acupressure and lower limb rehabilitation training machines can significantly reduce inflammation levels in patients. This may be because the specific stimulation of finger acupressure combined with lower limb rehabilitation training machines can affect the regulation of the autonomic nervous system, activate the parasympathetic nervous system, and the parasympathetic nervous system can inhibit the release of inflammatory factors (such as CRP) by regulating the vagus nerve (23, 24). The immune function and oxygenation status (SpO2) of patients in the treatment group were significantly better than those in the control group, which may be due to the improvement of blood circulation and the increase of oxygen content in the blood through lower limb rehabilitation training machines, thereby improving oxygenation status. The patient’s oxygenation status improves, which helps immune cells and immune factors to be delivered to the required sites faster, and the immune system is also enhanced accordingly. From the above analysis, it can be concluded that the combination of finger acupressure and lower limb rehabilitation training machine may improve the patient’s motor function and balance ability by promoting nerve repair, enhancing immune function, and reducing inflammatory response.

This study also found that, in addition to rehabilitation treatment methods, stroke type, stroke location, NIHSS score, and mRS score also affect the recovery of patients’ motor function and balance ability. Patients with ischemic stroke are more likely to recover, which may be because the occurrence of ischemic stroke is usually caused by blood flow interruption due to vascular obstruction. If timely treatment is given, such as mechanical thrombectomy, the interrupted blood flow can be restored, and some damaged brain tissue can also be restored. The occurrence area of this situation is limited, and other areas of the brain can compensate for the damaged area’s function through neural plasticity (25). With the help of rehabilitation training, patients can quickly recover their motor control and coordination abilities. However, hemorrhagic stroke involves a wider range, and the hematoma formed by bleeding may compress brain tissue, leading to nerve damage, and may also be accompanied by stronger inflammatory reactions and tissue necrosis, resulting in a slower recovery rate (26). The recovery speed of brainstem stroke is also slow, which may be because the brainstem is the connecting hub between the brain and the spinal cord, responsible for regulating various life activities, and the neural structure is complex. Therefore, once a stroke occurs, it will affect multiple basic life functions, making it difficult to recover (27). The higher the baseline NIHSS score and mRS score, the more difficult it is for patients to recover. This may be because the higher the score, the more severe the condition, the more significant the loss of neurological function, and may also be accompanied by the occurrence of various complications.

Interaction analysis found that the combination of finger acupressure and lower limb rehabilitation training machine had better recovery effects in patients with lower baseline NIHSS scores, mRS scores, and ischemic stroke. This indicates that our treatment method is more suitable for patients with milder conditions, who have stronger neural plasticity, less damage to the nervous system, and stronger self-regulation ability. This discovery is of great significance in providing more personalized rehabilitation treatment plans and achieving precise treatment.

Long term follow-up analysis found that the quality of life and recurrence rate of the treatment group were significantly better than those of the control group, which also indicates that the combination of finger acupressure and lower limb rehabilitation training machine can not only improve patients’ motor function and balance ability in the short term, but also have a positive impact on their long-term health status, further increasing the possibility of promoting this treatment plan in clinical practice.

Finger acupressure combined with lower limb rehabilitation training machines may cause adverse events such as muscle and joint discomfort, fatigue, skin damage, and mood swings during clinical application. Muscle and joint discomfort often occurs during the initial stages of treatment or when the intensity is too high, manifesting as localized soreness and limited mobility. Progressive loading training (initial intensity ≤ 60% of maximum tolerance) combined with post-training thermotherapy or low-frequency electrical stimulation can be adopted. Fatigue accumulation is related to training intensity and patient physical condition, and can be alleviated by following the principle of intermittent training (1:1 training-rest ratio) and enhancing nutritional support. There is a certain risk of skin damage at the acupoint stimulation site, and hydrogel pads and pressure-controllable straps can be used for protection. In case of injury, antibiotics can be applied externally in a timely manner. Patients may experience emotional reactions such as anxiety due to treatment pressure or expectations of treatment effects. Emotional regulation is achieved through psychological screening before training, environmental optimization, and cognitive behavioral intervention when necessary.

Our study also has certain limitations. Firstly, due to the retrospective design, there may be biases in data selection, and it is difficult to control potential confounding factors. Secondly, the study did not implement blinded assessment, which may introduce measurement bias. Thirdly, the small sample size (n = 80) limits the generalizability of the research results. Additionally, analysis based on non-publicly available data may be subject to selection bias. Finally, we were unable to conduct deeper mechanistic analysis, such as identifying the specific pathways through which finger acupressure combined with lower limb rehabilitation training improves patients’ motor function and balance ability. In the future, larger-scale prospective randomized controlled trials, blinded evaluation, and molecular assays (such as serum biomarker analysis, radiomics, etc.) are needed to further verify and improve the conclusions of this study.

## 5 Conclusion

This study found that finger acupressure combined with the lower limb rehabilitation machine can significantly improve motor function and balance in stroke patients by promoting neural repair, enhancing immune function, and reducing inflammation. The treatment is particularly effective in patients with lower baseline NIHSS and mRS scores and those with ischemic stroke. It also has positive effects on the long-term health status of patients.

## Data Availability

The raw data supporting the conclusions of this article will be made available by the authors, without undue reservation.
